# Pathogenic protease expression in murine OA is critically dependent upon mechanical joint loading

**DOI:** 10.1186/ar3674

**Published:** 2012-02-09

**Authors:** Annika Burleigh, Anastasios Chanalaris, Jeremy Saklatvala, Tonia Vincent

**Affiliations:** 1 Department of cell signalling, Kennedy Institute of Rheumatology, Oxford University, London W6 8LH, UK

## Background

Once considered a passive disease of 'wear and tear' of the joint, osteoarthritis (OA) is now known to be driven by the expression and activation of specific proteases that degrade the extracellular matrix of articular cartilage. Such proteases include aggrecanases, principally adistintegrin and metalloproteinase (Adamts) 4 and 5, and collagenases which are members of the matrix metalloproteinase (Mmp) family. In mice, Adamts5 and Mmp13 are considered to be the critical proteases in disease, as mice in which these proteases have been knocked out are protected from developing OA [1,2]. What drives these proteases in vivo is unknown, but one possibility is that mechanical factors alone are sufficient to lead to their expression and activation.

To test this hypothesis we investigated the effects of joint immobilisation on protease expression and the course of disease in mice with surgically induced OA.

## Materials and methods

Destabilisation of the medial meniscus or sham surgery was performed in 10 week old male mice. Joints were immobilised either by prolonged anaesthesia (for a max of 4 h to examine gene expression changes) or by sciatic neurectomy (for 4-6 h or for 12 weeks). mRNA was extracted from whole joints at 4-6 h following induction of OA. A microarray was performed and 47 genes validated by RT-PCR. Joints were examined histologically after 12 weeks forcartilage damage.

## Results

Many genes were regulated within 6 hours of OA surgery (compared to sham surgery) including Adamts5, Mmp3, IL1b, Ccl2, activin and TNF-stimulated gene 6 (Tsg6). Mmp13 was not regulated at this early time point. Of the 47 genes studied, all gene responses were strongly suppressed if the joint was immobilised (by prolonged anaesthesia). Joint immobilisation by sciatic neurectomy also suppressed a number of genes (approx. 50%) including Adamts5, and protected the joints from cartilage degradation at 12 weeks.

## Conclusion

Pathogenic protease expression occurs rapidly upon induction of OA in mice (within 6 h) and is highly mechanosensitive. Suppression of Adamts5 also occurs following sciatic neurectomy in which the joint is immobilised but the mice are able to bear weight (they walk with a 'splinted' knee). This suggests that dynamic flexion of the destabilised knee joint is important for induction of proteases and subsequent disease.The pathway by which joint cells sense and respond to these mechanical signals could represent a novel target for disease intervention.
